# Bis[(2*S*,4*S*)-4-(2-hy­droxy­eth­yl)-2-methyl­piperazine-1,4-diium] di-μ-chlorido-bis­[trichloridocadmium(II)]

**DOI:** 10.1107/S1600536811006945

**Published:** 2011-03-02

**Authors:** Tao Rong

**Affiliations:** a Ordered Matter Science Research Center, Southeast University, Nanjing 210096, People’s Republic of China

## Abstract

The asymmetric unit of the title compound, (C_7_H_18_N_2_O)_2_[Cd_2_Cl_8_], comprises one 4-(2-hy­droxy­eth­yl)-2-methyl­piperazine-1,4-diium dication and a half [Cd_2_Cl_8_]^4−^ anion. The two Cd atoms are each coordinated by two bridging Cl atoms and three terminal Cl atoms and the [Cd_2_Cl_8_]^4−^ anion is located on an inversion centre. The crystal structure consists of N—H⋯Cl hydrogen-bonded sheets, which are further linked by C—H⋯Cl contacts, yielding a three-dimensional network.

## Related literature

For general background to ferroelectric metal-organic frameworks, see: Fu *et al.* (2009 ▶, 2010 ▶); Ye *et al.* (2006 ▶); Zhang *et al.* (2008 ▶, 2010 ▶). 
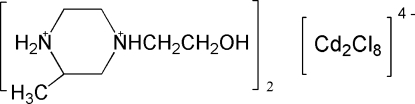


## Experimental

### 

#### Crystal data

(C_7_H_18_N_2_O)_2_[Cd_2_Cl_8_]
*M* *_r_* = 800.86
Monoclinic, 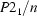

*a* = 8.0318 (16) Å
*b* = 11.144 (2) Å
*c* = 15.816 (3) Å
β = 97.81 (3)°
*V* = 1402.6 (5) Å^3^
*Z* = 2
Mo *K*α radiation
μ = 2.30 mm^−1^
*T* = 293 K
0.20 × 0.20 × 0.20 mm

#### Data collection

Rigaku SCXmini diffractometer
Absorption correction: multi-scan (*CrystalClear*; Rigaku, 2005 ▶) *T*_min_ = 0.632, *T*_max_ = 0.638
14193 measured reflections
3217 independent reflections
3008 reflections with *I* > 2σ(*I*)
*R*_int_ = 0.037

#### Refinement

*R*[*F* ^2^ > 2σ(*F* ^2^)] = 0.025
*wR*(*F* ^2^) = 0.060
*S* = 1.19
3217 reflections
136 parameters
2 restraints
H-atom parameters constrained
Δρ_max_ = 0.35 e Å^−3^
Δρ_min_ = −0.88 e Å^−3^

### 

Data collection: *CrystalClear* (Rigaku, 2005 ▶); cell refinement: *CrystalClear*; data reduction: *CrystalClear*; program(s) used to solve structure: *SHELXS97* (Sheldrick, 2008 ▶); program(s) used to refine structure: *SHELXL97* (Sheldrick, 2008 ▶); molecular graphics: *DIAMOND* (Brandenburg & Putz, 2005 ▶); software used to prepare material for publication: *SHELXL97*.

## Supplementary Material

Crystal structure: contains datablocks I, global. DOI: 10.1107/S1600536811006945/rn2080sup1.cif

Structure factors: contains datablocks I. DOI: 10.1107/S1600536811006945/rn2080Isup2.hkl

Additional supplementary materials:  crystallographic information; 3D view; checkCIF report

## Figures and Tables

**Table 1:** Hydrogen-bond geometry (Å, °)

*D*—H⋯*A*	*D*—H	H⋯*A*	*D*⋯*A*	*D*—H⋯*A*
O1—H1*A*⋯Cl4^i^	0.82	2.68	3.188 (3)	121
N1—H1*D*⋯Cl3^ii^	0.91	2.29	3.163 (3)	161
N2—H2*D*⋯Cl4^iii^	0.90	2.88	3.405 (3)	119
N2—H2*D*⋯Cl1^iv^	0.90	2.35	3.125 (3)	144
N2—H2*A*⋯Cl2^v^	0.90	2.25	3.119 (3)	164

Symmetry codes: (i) 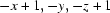; (ii) 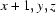; (iii) 
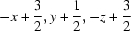
; (iv) 
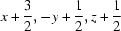
; (v) 
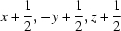
.

**Fig. 1.**
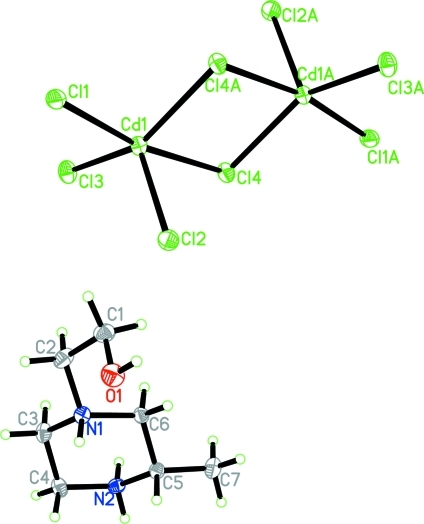
The molecular structure of the title compound, with the atomic numbering scheme. Displacement ellipsoids are drawn at the 30% probability level.[The suffix A denotes the symmetry code: -x - y 1 - z]

**Fig. 2.**
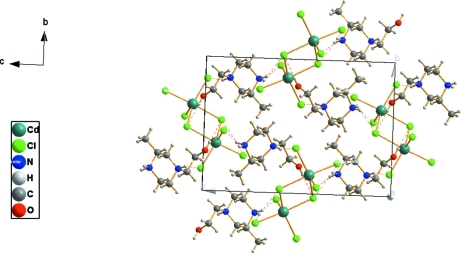
A view of the packing of the title compound, stacking along the a axis. Dashed lines indicate hydrogen bonds.

## supplementary crystallographic information

### Comment

The study of ferroelectric materials has received much attention. Some materials have predominantly dielectric-ferroelectric performance.The title compound was studied as part of our work to obtain potential ferroelectric phase-change materials [Fu *et al.*(2009); Ye *et al.* (2006); Zhang *et al.* (2008; 2010)] .

As one part of our continuing studies on dielectric-ferroelectric materials, we synthesized the title compound (C_7_H_18_N_2_O).CdCl_4_ (Fig 1)_._Unfortunately, the study carried out on the title compound indicated that the permittivity is temperature-independent, suggesting that there may be no dielectric disuniformity between 80 K to 350 K [Fu *et al.* (2010)].

The asymmetric unit of the title compound contains one [C_7_H_17_N_2_O] ^2+^ basic ion and half of the [Cd_2_Cl_8_]^4-^ complex ion which is situated on an inversion centre. The intermolecular hydrogen bonds (O1—H1A···Cl4, N1—H1D···Cl3, N2—H2D···Cl4, N2—H2D···Cl1 and N2—H2A···Cl2) link the molecules into sheets and stabilize the structure (Fig 2).

### Experimental

Ethylene oxide (25 mmol) was added by bubbling of this gas into a solution of rac-2-methyl piperazine (10 mmol) in toluene at 318–323 K. The toluene solvent was then removed under reduced pressure, the rac-2-methyl-4-ethoxyl piperazine was obtained at 376–381 K by reduced pressure distillation of the mixture. A solution of chlorhydric acid (10 mmol) was added to a solution of half equimolar amount of rac-2-methyl-4-ethoxyl piperazine inethanol (20 mL), then cadmium chloride(5 mmol) in water (10 mL) was added. Crystals suitable for structure determination were grown by slow evaporation of the mixture at room temperature

### Refinement

Positional parameters of all the H atoms bonded to C atoms were calculated geometrically and were allowed to ride on the C atoms to which they are bonded, with *U*_iso_(H) = 1.2*U*_eq_(C) and *U*_iso_(H) = 1.5*U*_eq_(C) for the methyl group. The other H bonded to O/N atoms were calculated geometrically and were allowed to ride on the O/N atoms with *U*_iso_(H) = 1.2*U*_eq_(N) and *U*_iso_(H) = 1.5*U*_eq_(O).

### Crystal data

**Table:**

(C_7_H_18_N_2_O)_2_[Cd_2_Cl_8_]	*Z* = 2
*M**_r_* = 800.86	*F*(000) = 792
Monoclinic, *P*2_1_/*n*	*D*_x_ = 1.896 Mg m^−^^3^
Hall symbol: -P 2yn	Mo *K*α radiation, λ = 0.71073 Å
*a* = 8.0318 (16) Å	θ = 3.0–27.5°
*b* = 11.144 (2) Å	µ = 2.30 mm^−^^1^
*c* = 15.816 (3) Å	*T* = 293 K
β = 97.81 (3)°	Prism, colourless
*V* = 1402.6 (5) Å^3^	0.20 × 0.20 × 0.20 mm

### Data collection

**Table:**

Rigaku SCXmini diffractometer	3217 independent reflections
Radiation source: fine-focus sealed tube	3008 reflections with *I* > 2σ(*I*)
graphite	*R*_int_ = 0.037
Detector resolution: 13.6612 pixels mm^-1^	θ_max_ = 27.5°, θ_min_ = 3.0°
CCD_Profile_fitting scans	*h* = −10→10
Absorption correction: multi-scan (*CrystalClear*; Rigaku, 2005)	*k* = −14→14
*T*_min_ = 0.632, *T*_max_ = 0.638	*l* = −20→20
14193 measured reflections	

### Refinement

**Table:**

Refinement on *F*^2^	Primary atom site location: structure-invariant direct methods
Least-squares matrix: full	Secondary atom site location: difference Fourier map
*R*[*F*^2^ > 2σ(*F*^2^)] = 0.025	Hydrogen site location: inferred from neighbouring sites
*wR*(*F*^2^) = 0.060	H-atom parameters constrained
*S* = 1.19	*w* = 1/[σ^2^(*F*_o_^2^) + (0.022*P*)^2^ + 0.433*P*] where *P* = (*F*_o_^2^ + 2*F*_c_^2^)/3
3217 reflections	(Δ/σ)_max_ = 0.001
136 parameters	Δρ_max_ = 0.35 e Å^−^^3^
2 restraints	Δρ_min_ = −0.88 e Å^−^^3^

### Special details

**Table:**

Refinement. Refinement of *F*^2^ against ALL reflections. The weighted *R*-factor *wR* and goodness of fit *S* are based on *F*^2^, conventional *R*-factors *R* are based on *F*, with *F* set to zero for negative *F*^2^. The threshold expression of *F*^2^ > σ(*F*^2^) is used only for calculating *R*-factors(gt) *etc*. and is not relevant to the choice of reflections for refinement. *R*-factors based on *F*^2^ are statistically about twice as large as those based on *F*, and *R*- factors based on ALL data will be even larger.

### Fractional atomic coordinates and isotropic or equivalent isotropic displacement parameters (Å^2^)

**Table:**

	*x*	*y*	*z*	*U*_iso_*/*U*_eq_	
Cd1	0.07749 (3)	0.14056 (2)	0.435513 (16)	0.02832 (11)	
Cl4	0.13747 (12)	0.02958 (8)	0.59105 (5)	0.0299 (2)	
Cl2	0.34061 (12)	0.04831 (9)	0.39588 (6)	0.0364 (2)	
Cl3	0.13624 (14)	0.33698 (9)	0.51000 (6)	0.0363 (2)	
Cl1	−0.02934 (12)	0.23097 (9)	0.28873 (6)	0.0369 (2)	
N2	1.0951 (4)	0.3458 (3)	0.78336 (19)	0.0267 (6)	
H2A	1.0310	0.3654	0.8237	0.032*	
H2D	1.2013	0.3360	0.8091	0.032*	
N1	0.8525 (4)	0.3466 (3)	0.63036 (19)	0.0262 (6)	
H1D	0.9222	0.3270	0.5917	0.031*	
C6	0.8591 (5)	0.2469 (3)	0.6942 (2)	0.0266 (7)	
H6A	0.8235	0.1729	0.6649	0.032*	
H6B	0.7812	0.2642	0.7344	0.032*	
C4	1.0905 (5)	0.4452 (3)	0.7205 (2)	0.0332 (8)	
H4A	1.1679	0.4280	0.6800	0.040*	
H4B	1.1262	0.5191	0.7499	0.040*	
C5	1.0336 (5)	0.2302 (3)	0.7425 (2)	0.0261 (7)	
H5A	1.1095	0.2063	0.7019	0.031*	
C3	0.9163 (5)	0.4608 (3)	0.6735 (2)	0.0328 (8)	
H3A	0.8411	0.4854	0.7134	0.039*	
H3B	0.9173	0.5236	0.6311	0.039*	
O1	0.7146 (4)	0.2021 (3)	0.49063 (18)	0.0422 (7)	
H1A	0.6727	0.1408	0.4683	0.063*	
C1	0.6018 (5)	0.2547 (4)	0.5418 (3)	0.0375 (9)	
H1B	0.5787	0.1985	0.5857	0.045*	
H1C	0.4965	0.2739	0.5068	0.045*	
C7	1.0337 (6)	0.1329 (4)	0.8089 (3)	0.0410 (10)	
H7A	1.1452	0.1236	0.8388	0.061*	
H7B	0.9976	0.0587	0.7815	0.061*	
H7C	0.9584	0.1546	0.8486	0.061*	
C2	0.6798 (5)	0.3669 (4)	0.5825 (3)	0.0372 (9)	
H2B	0.6075	0.3986	0.6215	0.045*	
H2C	0.6868	0.4265	0.5384	0.045*	

### Atomic displacement parameters (Å^2^)

**Table:**

	*U*^11^	*U*^22^	*U*^33^	*U*^12^	*U*^13^	*U*^23^
Cd1	0.03076 (17)	0.02561 (17)	0.02899 (17)	−0.00180 (10)	0.00547 (12)	0.00126 (10)
Cl4	0.0366 (5)	0.0285 (5)	0.0240 (4)	−0.0056 (4)	0.0020 (3)	0.0008 (3)
Cl2	0.0334 (5)	0.0380 (5)	0.0409 (5)	0.0029 (4)	0.0160 (4)	0.0066 (4)
Cl3	0.0476 (6)	0.0283 (5)	0.0354 (5)	−0.0012 (4)	0.0140 (4)	−0.0020 (4)
Cl1	0.0296 (5)	0.0426 (6)	0.0379 (5)	−0.0037 (4)	0.0026 (4)	0.0134 (4)
N2	0.0242 (15)	0.0290 (16)	0.0268 (15)	0.0015 (12)	0.0028 (12)	−0.0039 (12)
N1	0.0293 (16)	0.0260 (16)	0.0234 (15)	0.0015 (12)	0.0037 (12)	−0.0019 (12)
C6	0.0281 (18)	0.0222 (18)	0.0298 (18)	−0.0016 (14)	0.0056 (14)	0.0004 (14)
C4	0.038 (2)	0.0281 (19)	0.0325 (19)	−0.0086 (16)	0.0027 (16)	0.0004 (16)
C5	0.0274 (18)	0.0234 (18)	0.0279 (17)	0.0014 (14)	0.0049 (14)	−0.0028 (14)
C3	0.043 (2)	0.0246 (19)	0.0294 (19)	−0.0022 (16)	−0.0009 (16)	0.0004 (15)
O1	0.0465 (17)	0.0395 (17)	0.0403 (16)	−0.0039 (13)	0.0052 (13)	−0.0102 (13)
C1	0.031 (2)	0.040 (2)	0.039 (2)	0.0002 (17)	−0.0041 (17)	−0.0033 (18)
C7	0.049 (3)	0.031 (2)	0.042 (2)	0.0033 (18)	0.003 (2)	0.0055 (18)
C2	0.038 (2)	0.034 (2)	0.037 (2)	0.0083 (17)	−0.0064 (18)	−0.0045 (17)

### Geometric parameters (Å, °)

**Table:**

Cd1—Cl3	2.5003 (11)	C4—C3	1.502 (6)
Cd1—Cl2	2.5052 (11)	C4—H4A	0.9700
Cd1—Cl4^i^	2.5603 (10)	C4—H4B	0.9700
Cd1—Cl1	2.5690 (11)	C5—C7	1.509 (5)
Cd1—Cl4	2.7371 (10)	C5—H5A	0.9800
Cl4—Cd1^i^	2.5603 (10)	C3—H3A	0.9700
N2—C4	1.486 (5)	C3—H3B	0.9700
N2—C5	1.495 (4)	O1—C1	1.421 (5)
N2—H2A	0.9000	O1—H1A	0.8200
N2—H2D	0.9000	C1—C2	1.503 (5)
N1—C6	1.497 (4)	C1—H1B	0.9700
N1—C3	1.501 (5)	C1—H1C	0.9700
N1—C2	1.505 (5)	C7—H7A	0.9600
N1—H1D	0.9100	C7—H7B	0.9600
C6—C5	1.514 (5)	C7—H7C	0.9600
C6—H6A	0.9700	C2—H2B	0.9700
C6—H6B	0.9700	C2—H2C	0.9700
			
Cl3—Cd1—Cl2	111.44 (4)	C3—C4—H4B	109.5
Cl3—Cd1—Cl4^i^	143.67 (4)	H4A—C4—H4B	108.1
Cl2—Cd1—Cl4^i^	103.18 (4)	N2—C5—C7	110.4 (3)
Cl3—Cd1—Cl1	95.79 (4)	N2—C5—C6	109.9 (3)
Cl2—Cd1—Cl1	97.14 (4)	C7—C5—C6	110.7 (3)
Cl4^i^—Cd1—Cl1	90.41 (4)	N2—C5—H5A	108.6
Cl3—Cd1—Cl4	88.47 (3)	C7—C5—H5A	108.6
Cl2—Cd1—Cl4	89.31 (4)	C6—C5—H5A	108.6
Cl4^i^—Cd1—Cl4	81.10 (4)	N1—C3—C4	111.4 (3)
Cl1—Cd1—Cl4	170.34 (3)	N1—C3—H3A	109.4
Cd1^i^—Cl4—Cd1	98.90 (4)	C4—C3—H3A	109.4
C4—N2—C5	112.1 (3)	N1—C3—H3B	109.4
C4—N2—H2A	109.2	C4—C3—H3B	109.4
C5—N2—H2A	109.2	H3A—C3—H3B	108.0
C4—N2—H2D	109.2	C1—O1—H1A	109.5
C5—N2—H2D	109.2	O1—C1—C2	109.0 (3)
H2A—N2—H2D	107.9	O1—C1—H1B	109.9
C6—N1—C3	110.1 (3)	C2—C1—H1B	109.9
C6—N1—C2	113.4 (3)	O1—C1—H1C	109.9
C3—N1—C2	109.6 (3)	C2—C1—H1C	109.9
C6—N1—H1D	107.9	H1B—C1—H1C	108.3
C3—N1—H1D	107.9	C5—C7—H7A	109.5
C2—N1—H1D	107.9	C5—C7—H7B	109.5
N1—C6—C5	112.2 (3)	H7A—C7—H7B	109.5
N1—C6—H6A	109.2	C5—C7—H7C	109.5
C5—C6—H6A	109.2	H7A—C7—H7C	109.5
N1—C6—H6B	109.2	H7B—C7—H7C	109.5
C5—C6—H6B	109.2	C1—C2—N1	113.1 (3)
H6A—C6—H6B	107.9	C1—C2—H2B	109.0
N2—C4—C3	110.8 (3)	N1—C2—H2B	109.0
N2—C4—H4A	109.5	C1—C2—H2C	109.0
C3—C4—H4A	109.5	N1—C2—H2C	109.0
N2—C4—H4B	109.5	H2B—C2—H2C	107.8
			
Cl3—Cd1—Cl4—Cd1^i^	145.07 (4)	N1—C6—C5—N2	55.2 (4)
Cl2—Cd1—Cl4—Cd1^i^	−103.46 (4)	N1—C6—C5—C7	177.4 (3)
Cl4^i^—Cd1—Cl4—Cd1^i^	0.0	C6—N1—C3—C4	55.9 (4)
Cl1—Cd1—Cl4—Cd1^i^	28.7 (2)	C2—N1—C3—C4	−178.7 (3)
C3—N1—C6—C5	−55.7 (4)	N2—C4—C3—N1	−56.5 (4)
C2—N1—C6—C5	−178.9 (3)	O1—C1—C2—N1	−53.4 (5)
C5—N2—C4—C3	56.5 (4)	C6—N1—C2—C1	−52.8 (5)
C4—N2—C5—C7	−177.7 (3)	C3—N1—C2—C1	−176.3 (3)
C4—N2—C5—C6	−55.3 (4)		

Symmetry codes: (i) −*x*, −*y*, −*z*+1.

### Hydrogen-bond geometry (Å, °)

**Table:**

*D*—H···*A*	*D*—H	H···*A*	*D*···*A*	*D*—H···*A*
O1—H1A···Cl4^ii^	0.82	2.68	3.188 (3)	121
N1—H1D···Cl3^iii^	0.91	2.29	3.163 (3)	161
N2—H2D···Cl4^iv^	0.90	2.88	3.405 (3)	119
N2—H2D···Cl1^v^	0.90	2.35	3.125 (3)	144
N2—H2A···Cl2^vi^	0.90	2.25	3.119 (3)	164

Symmetry codes: (ii) −*x*+1, −*y*, −*z*+1; (iii) *x*+1, *y*, *z*; (iv) −*x*+3/2, *y*+1/2, −*z*+3/2; (v) *x*+3/2, −*y*+1/2, *z*+1/2; (vi) *x*+1/2, −*y*+1/2, *z*+1/2.
